# Singlet Oxygen Production by PSII Under Light Stress: Mechanism, Detection and the Protective role of β-Carotene

**DOI:** 10.1093/pcp/pcu040

**Published:** 2014-03-22

**Authors:** Alison Telfer

**Affiliations:** Department of Life Sciences, Sir Ernst Chain Building, Imperial College London, London SW7 2AZ, UK

**Keywords:** Chlorophyll, Photosynthesis, Reactive oxygen species, Triplet states

## Abstract

In this review, I outline the indirect evidence for the formation of singlet oxygen (^1^O_2_) obtained from experiments with the isolated PSII reaction center complex. I also review the methods we used to measure singlet oxygen directly, including luminescence at 1,270 nm, both steady state and time resolved. Other methods we used were histidine-catalyzed molecular oxygen uptake (enabling ^1^O_2_ yield measurements), and dye bleaching and difference absorption spectroscopy to identify where quenchers of ^1^O_2_ can access this toxic species. We also demonstrated the protective behavior of carotenoids bound within Chl–protein complexes which bring about a substantial amount of ^1^O_2_ quenching within the reaction center complex. Finally, I describe how these techniques have been used and expanded in research on photoinhibition and on the role of ^1^O_2_ as a signaling molecule in instigating cellular responses to various stress factors. I also discuss the current views on the role of ^1^O_2_ as a signaling molecule and the distance it might be able to travel within cells.

## Introduction

Singlet oxygen (^1^O_2_) is an electronically excited state of molecular oxygen which is extremely reactive (Ogilby 2010). It attacks and oxidizes proteins, lipids and nucleic acids, and consequently it is an important reactive oxygen species (ROS) in biological systems. It is less stable than triplet oxygen (^3^O_2_), and may be formed in a variety of ways; however, a common way is by electronic energy transfer from the triplet state of a photosensitized pigment or dye molecule.
(1)
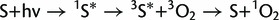

where S is a sensitizer molecule, dye or pigment. During oxygenic photosynthesis (Blankenship 2014), ^1^O_2_ is easily formed as Chl molecules are very good photosensitizers and the nature of the photosynthetic process means that there is always plenty of ground state, ^3^O_2_, around.

Photosensitization of the triplet state of Chl leads to formation of ^1^O_2_ unless Chl triplets are removed rapidly before ^1^O_2_ formation can take place (Ogilby 2010). Carotenoid (Car) molecules are very effective quenchers of triplet Chl (Frank and Cogdell 1996) and also directly of ^1^O_2_ (Hirayama et al. 1994) in photosynthetic systems. However, despite their effectiveness in the protection of photosynthetic organisms, high light intensities do bring about loss of photosynthetic activity in oxygenic organisms as reflected by the physiological phenomenon of photoinhibition (Prasil et al. 1992, Aro et al. 1993, Adir et al. 2003).

The phenomenon of photoinhibition has been localized mainly to the photosynthetic reaction center (RC) of PSII. High light initially causes a decrease in the rate of electron transport through PSII and preferential degradation of the Dl protein, an intrinsic subunit of the complex. Restoration of activity requires de novo protein synthesis. Molecular oxygen has been implicated in photoinhibition (Prasil et al. 1992), and damaging oxygen species, ^1^O_2_ and other ROS, are likely to be the agents that activate Dl protein degradation (Barber and Andersson 1992, Fischer et al. 2013). Keren et al. (1997) have also argued that PSII can be inactivated at low light levels and that formation of the Chl triplet state in PSII and ^1^O_2_ is involved. This is discussed in more detail later.

Here I will describe the history of the detection of ^1^O_2_ formed by isolated photosynthetic complexes and demonstrate the protective behavior of Cars bound within Chl–protein complexes, and then relate this information to current research in photoinhibition and its function as a signaling molecule in instigating cellular responses to various stress factors.

## Photosynthetic Pigment–Protein complexes

Photosynthetic electron transport is carried out by a series of Chl–protein complexes. The antenna pigments are bound to light-harvesting pigment–protein complexes (LHCI and LHCII), which absorb light, producing the first excited singlet state of Chl, and then there are a series of energy transfer reactions, between the antenna Chl and the RCs. Here the first photochemical step occurs in which a specialized Chl molecule (P) on excitation to its excited singlet state passes on an electron to an acceptor molecule (A) to form the primary radical pair, P^+^A^–^. During oxygenic photosynthesis, two photochemical reactions occur in series catalyzed by two pigment–protein complexes known as PSII and PSI. In PSII, the oxidized electron donor is re-reduced by electrons extracted from water (a by-product being molecular oxygen after extraction of four electrons from two water molecules), while in PSI the reduced acceptor donates two electrons to NADP^+^ to form NADPH.

The four pigment–protein complexes of green plants (PSI, PSII, and the antenna complexes LHCI and LHCII) all bind approximately 1 Car molecule per 4 Chl molecules and it is the Cars that normally prevent the formation of ^1^O_2_. Car-deficient mutants can grow from seed but are bleached and die as soon as they see normal light (Walles 1965).

**Fig. 1:**
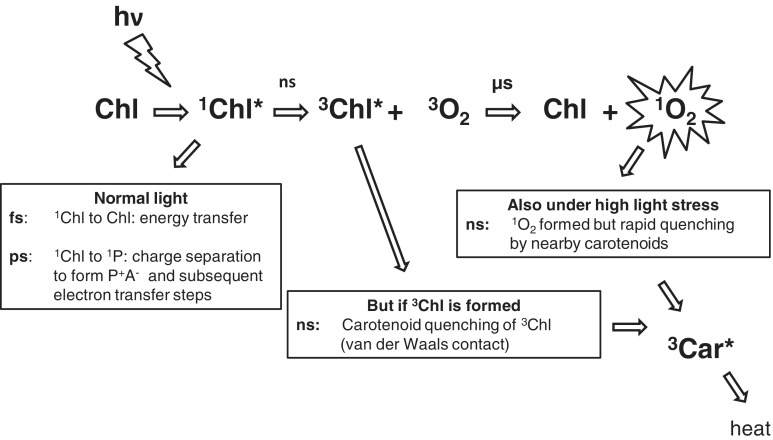
Avoidance of ^3^Chl and ^1^O_2_ formation: time scales involved.

Car molecules, provided they are bound within van der Waals distance of the Chl, are extremely efficient quenchers of Chl triplets (Fig. 1). One of the earliest experiments demonstrating the transfer of energy from the triplet excited state of Chl to Car was the so-called ‘valve reaction’ of Witt (1971) in which an increase in the size of an absorbance change (due to ^3^Car formation) was seen only once photosynthetic electron transfer was light saturated. It then continued to rise more or less linearly with the intensity of the laser flash energy.

There are two mechanisms by which Chl triplets are formed. In the antenna complexes it is by intersystem crossing:
(2)
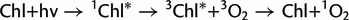

while in the PSII reaction centre it is by the radical pair (RP) mechanism:
(3)
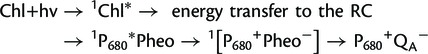

P_680_^+^Q_A_^–^ recombination occurs either directly or indirectly:
(4)
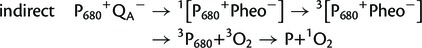

(5)
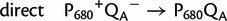

where P_680_ is the primary Chl electron donor in PSII, Pheo is the primary electron acceptor in PSII, and Q_A_ is a plastoquinone molecule, which is the second electron acceptor in PSII. The indirect pathway leads to formation of ^1^O_2_ while the direct pathway does not. In experiments where the midpoint potential of the secondary electron acceptor Q_A_ was made more positive (and hence decreased the likelihood of the indirect pathway), the yield of ^1^O_2_ was lowered while when it was made more negative the yield was increased (Krieger-Liszkay and Rutherford 1998, Fufezan et al. 2002).

The two mechanisms of Chl triplet formation can be distinguished by their electron paramagneitc resonance (EPR) signal properties. The radical pair triplet is only formed after formation of P_680_^+^Pheo^–^, which gives a spin-polarized EPR triplet signal, after spin dephasing, which has a characteristic absorption (A) and emission (E) spectrum (AEEAAE) as opposed to the pattern seen in triplets formed by intersystem crossing (AEAEAE) (van Mieghem et al. 1991).

## The D1D2 Reaction Center Complex of PSII and Indirect Evidence for ^1^O_2_ Formation

**Fig. 2:**
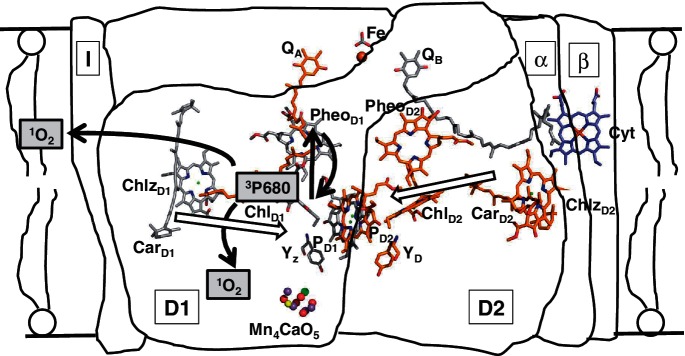
Schematic diagram of the electron transfer reactions occurring in the membrane-bound PSII RC and the formation of singlet oxygen at the site of ^3^P680. The purified complex (D1, D2, α and β subunits of Cyt *b*_559_ and the PsbI protein) has lost both of the secondary electron acceptors, Q_A_ and Q_B_, the non-heme iron (Fe) and also the water-splitting Mn cluster, Mn_4_CaO_5_. The figure shows that if the triplet state of P_680_ is formed it will be quenched by ground state oxygen to form ^1^O_2_ which can damage either the pigment–protein complex or the lipid membrane. The cofactor arrangement in the RC complex shows the distance of the two β-carotenes from the four central Chl cofactors, based on Umena et al. (2011).

It was the instability, in the presence of molecular oxygen, of the PSII RC complex (also known as the D1D2 complex) isolated by Satoh and colleagues (Nanba and Satoh 1987) which first led to the suggestion that large amounts of ^1^O_2_ were being formed by this complex due to interaction of the radical pair triplet state with molecular oxygen (Barber et al. 1987, Durrant et al. 1990) (see Fig. 2).

The presence of molecular oxygen was found to bleach the sample and to inactivate P_680_, the primary electron donor, it also shortened the P_680_ triplet lifetime from 1 ms (under anaerobic conditions) to 33 µs (Durrant et al. 1990). The first observation was that the complex showed a high yield of the radical pair recombination triplet state (Okamura et al. 1987), which shows the distinctive and specific absorption and emission EPR spectrum (AEEAAE), indicative of the formation of the P_680_ triplet state via the RP mechanism (see Equations 3–5). There was virtually no triplet Car formed by the D1D2 RC complex (Takahashi et al. 1987, Durrant et al. 1990).

The question arose as to why the two Cars bound to the RC were not protecting against ^1^O_2_ formation by quenching the RP triplet (Telfer 2002). This was shown by De Las Rivas et al. (1993) to be because the Car is oxidized by P_680_^+^ if this highly oxidizing species is allowed to persist for any length of time, i.e. in the presence of an added artificial electron acceptor which can stabilize P_680_^+^ (Barber et al. 1987). The oxidized Car is very unstable and its absorption (420–520 nm) is rapidly irreversibly bleached (De Las Rivas et al. 1993).

## ^1^O_2_ Production by Photosynthetic Pigment–Protein Complexes

Conditions arise where ^1^O_2_ can be formed in photosynthetic light-harvesting antenna complexes by triplet–triplet excitation transfer (intersystem crossing), as was seen by Wolff and Witt (1969) at high light intensities, when electron transport is light saturated and the Car triplet yield is increased substantially. Here Cars can prevent ^1^O_2_ formation as they are bound in the antenna complexes within van der Waals contact and so quench Chl triplets which are formed on a nanosecond time scale (Schödel et al. 1998). The Car triplets then decay harmlessly, releasing heat.

It is very unlikely that Chl triplets are formed in the PSI RC under photoinhibitory conditions (Hideg and Vass 1995, Rutherford et al. 2012), but there is evidence for their formation in PSII which is related to the very high oxidizing potential of P_680_^+^, which is required for water oxidation to occur. The β-carotene in the RC has been shown to be bound well beyond van der Waals distance from the Chls of the RC cofactor cluster (Fig. 2; Loll et al. 2005) and so cannot be invoked to quench directly any ^3^P_680_ which might be formed. There is strong evidence that both under high light (van Mieghem et al. 1989, Vass et al. 1992) and also under very low light ^3^P_680_ is formed via the radical pair recombination pathway (Keren et al. 1997). The significance of this mechanism for formation of the primary donor triplet state is that P_680_^+^ must be formed first (Equations 3–5) and as it has such a high redox potential any Car bound close enough to quench a triplet would have been oxidized previously by the cationic P_680_.

It was not only the very high sensitivity of the isolated D1D2 complex to light in the presence of molecular oxygen which suggested that ^1^O_2_ was being formed at a high yield. The lifetime of the ^3^P_680_ was lengthened dramatically under anaerobic conditions (from ∼30 µs to 1 ms) and the consequent inactivation of P_680_, loss of the red-most absorbance due to P_680_ and degradation of the D1 and D2 proteins were ascribed to the damaging effect of ^1^O_2_ (Durrant et al. 1990, Barber and Archer 2001). Note that during the early 1990s it became clear that ^1^O_2_ formation by the isolated D1D2 complex occurs but it had only been detected by indirect methods.

## Direct Detection of ^1^O_2_ Formed by Isolated PSII RCs

It was in the early 1990s that we began using the techniques used by experimentalists studying photosensitization of ^1^O_2_ by dyes to be used in photodynamic therapy (PDT) (Allison 2004) to look for direct evidence for ^1^O_2_ formation by the D1D2 complex. The dramatic change in the lifetime of the P_680_ triplet and the fact that the Chl triplet yield was very high, about 0.3, whereas that of the Car triplet was very low (0.03) all suggested that the yield of ^1^O_2_ should also be high (Durrant et al. 1990).

The first technique we used was to look for the very weak luminescence at 1,270 nm from ^1^O_2_. This is so weak that it is only detected using a 77 K cooled photomultiplier (Macpherson et al. 1993). Here we showed steady-state emission of luminescence at 1,270 nm from the isolated D1D2 complex on illumination under aerobic conditions. The luminescence (L_1,270_) was partially quenched by azide, a known quencher of ^1^O_2_. The azide-quenchable part of the signal (30–50%) was concluded to be due to ^1^O_2_ and the remaining part to infrared phosphorescence from the Chls in the PSII RC. Note that it was necessary to exchange the RCs into a D_2_O medium as water itself is a very good quencher of ^1^O_2_, shortening its lifetime from ∼70 µs to ∼3 µs and hence reducing the size of the steady-state emission signal until it was undetectable (Gorman and Rogers 1989, Wilkinson et al. 1995). As concluded by Telfer et al. (1994a), this was probably the first direct observation of ^1^O_2_ luminescence sensitized by an intrinsically bound chromophore in a defined biological system as opposed to a sensitizer-doped biological material (e.g. Firey and Rogers 1998).

Complementary to the L_1,270_ method, we also used a chemical trapping technique to estimate the yield of ^1^O_2_ formed on illumination of the isolated D1D2 complex (Telfer et al. 1994a). The uptake of molecular oxygen due to the reaction of ^1^O_2_ with histidine or imidazole was measured using an oxygen electrode, and the yield was compared with similar experiments using ^1^O_2_ sensitizing dyes such as mesotetra-(4-sulfonatophenyl)porphine (TPPS) and aluminum phthalocyanine disulfonate (AlPcS) for which the ^1^O_2_ yield is already known. We found that the yield of ^1^O_2_ was about 0.16 whereas the yield of ^3^P_680_ in the complex is 0.3 (Durrant et al. 1990). The lower yield of ^1^O_2_ as compared with that of the Chl triplet is to be expected as some ^1^O_2_ will be quenched rapidly by the protein and pigments within the RC complex before it escapes into the medium.

We also used the dye bleaching technique of Kraljic and El Mohsni (1978) to detect ^1^O_2_. This technique is based on the bleaching of *p*-nitrosodimethylaniline (RNO) to the nitro form caused by the trans-annular peroxide product of the reaction of ^1^O_2_ with either histidine or imidazole. We measured the bleaching of the dye due to ^1^O_2_ simultaneously with the bleaching of Chl associated with the inactivation of the sample by this ROS (Telfer et al. 1994a).

All these techniques indicated that under illumination and aerobic conditions, the D1D2 complex produces a large amount of ^1^O_2_. It escapes from the complex into the aqueous medium, and we conclude that it was quenched or detected there as there was no protection against bleaching of the Chl by added quenchers such as azide or histidine or by water vs. D_2_O (Telfer et al. 1994a). This effect had been noted by Macpherson et al. (1993) where the bleaching of Chl was not prevented by the addition of azide, although the L_1,270_ was quenched, leading us to conclude that the ^1^O_2_ detected as emission at 1,270 nm is in a different environment (accessible to quenchers) from that giving rise to the beaching of Chl. In essence the ^1^O_2_ is formed within the D1D2 complex on the Chl of P_680_ and then it diffuses out of the complex into the aqueous medium where not only is it accessible to water-soluble quenchers such as azide but its lifetime is lengthened by the presence of D_2_O as compared with H_2_O. In experiments where H_2_O and D_2_O buffers were compared, there was no stimulation of the inactivation of P_680_ in the latter medium compared with H_2_O medium. This indicates that several rounds of buffer exchange of the complex (using Millipore concentrator tubes; see Macpherson et al. 1993), which was originally isolated in an H_2_O-based medium, into a D_2_O-based medium either does not exchange the water molecules within the complex or that there are no water molecules close enough to P_680_ to quench the ^1^O_2_. The latest structure of the PSII core complex, which is at 1.9 Å resolution, shows that the majority of the very many water molecules in the structure are located in two layers on the surfaces of the stromal and lumenal sides (Umena et al. 2011). Of the few water molecules found in the interior of the complex, most of them serve as ligands to Chls. Note that the magnesium of the accessory Chl on D1, which is thought to be where the ^3^P_680_ is located, is liganded by a water molecule as is that of accessory Chl_D2_.

This evidence that the site of ^1^O_2_ formation is deep within the D1D2 complex was confirmed by Telfer et al. (1994a) in RNO bleaching experiments. Absorption difference spectra show clearly that although the quencher, azide, prevents the RNO bleaching it does not stop the loss of absorption of P_680_, which we concluded is caused by ^1^O_2_ before it escapes from the complex, i.e. internal intrinsic quenching mechanisms compete very effectively with externally added water-soluble quenchers. However, the presence of D_2_O in place of water increased the rate of RNO bleaching approximately 3-fold, which is consistent with the increase in the lifetime of ^1^O_2_ in the external medium when it is present in place of H_2_O.

Telfer et al. (1994a) also carried out a number of experiments to show that it was ^1^O_2_ causing the inactivation of P_680_ and that it was not due to any other ROS. As Foote (1990) warned, ‘detection of a species does not indicate its intermediacy in a process’, and in PDT it has been difficult to demonstrate that it is actually ^1^O_2_ causing cell death. We definitively showed that it is ^1^O_2_ that brings about Chl bleaching and inactivation of P_680_ in the isolated D1D2 complex (Telfer et al. 1994a).

## Correlation of P_680_ Triplet Decay and L_1,270_ Signal Rise Rates in the Isolated PSII RC

We also measured time-resolved 1,270 nm luminescence of ^1^O_2_, formed on illumination of the D1D2 complex in the presence of D_2_O when suspended in air-saturated medium (Macpherson et al. 1993, Telfer et al. 1994b), and later correlated the rise time of the L_1,270_ signal with the decay of the ^3^P_680_ absorption decrease at 680 nm (Telfer et al. 1999). Here we showed the similarity in the triplet decay rate and L_1,270_ rise times and the dependence on the molecular oxygen concentration for the rate of quenching of the triplet and rise of the L_1,270_ which indicated that ^1^O_2_ is formed directly by quenching of ^3^P_680_. Li et al. (2012) carried out similar experiments, measuring time-resolved L_1,270_, on isolated PSII RCs in aqueous media and concluded that the lifetime of the ^1^O_2_ would be so short (<0.5 µs) that determining the ^1^O_2_ rate constants in chloroplasts suspended in aqueous medium, i.e. in vivo conditions, would be ‘a tall order’, i.e. they imply it would be impossible.

## Role of β-Carotene in Protection against Photodamage

As shown in Fig. 2, the β-carotene bound to the PSII RC can act as an admittedly relatively inefficient electron donor to P_680_^+^ (De Las Rivas et al. 1993). This occurs if the lifetime of the oxidized donor is prolonged by the addition of an artificial electron acceptor, e.g. silicomolybdate and dibromothymoquinone, which are able to accept electrons directly from the pheophytin primary electron acceptor which is bound to the D1 protein of the RC complex. In addition to this role of rereduction of P_680_^+^ (if it is not reduced rapidly by the tyrosine electron donor, Y_Z_, and then by electrons from water), the Car should also quench ^1^O_2_, diffusing within the Chl protein complex, directly. The idea, as discussed already, is that the Car cannot be bound closely enough to quench ^3^P_680_ directly as it would be oxidized first by P_680_^+^ which has to be formed prior to the formation of the triplet by the radical pair mechanism (Telfer 2002). Indeed the crystal structure of PSII by Loll et al. (2005) shows that the closest approach of the two β-carotenes is 13.2 Å for Car_D2_ to Chl_D2_ and 19.9 Å for Car_D1_ to Chl_D1_ (Fig. 2). Using the Moser and Dutton rule (1992), both distances are far too great to allow either rapid quenching of ^3^P_680_ or rapid electron transfer directly to P_680_^+^.

The question arises as to what is the role of the Cars bound to the PSII RC aside from rereduction of any stabilized P_680_^+^. As discussed earlier, it is inevitable that some ^3^P_680_ will be formed during turnover of the PSII RC (van Mieghem et al. 1989, Keren et al. 1997) and, because there will always be molecular oxygen around, ^1^O_2_ will be formed. It was known that carotenoids can quench ^1^O_2_ directly (Hirayama et al. 1994) and so we tested the proposition that this is another role for the Cars in the RC. The β-carotene level of isolated PSII RCs was lowered by extensive washing of the preparatory anion exchange column with low salt buffer before elution with high salt, and thus we prepared complexes with various levels of Car. We were then able to show an inverse correlation between the size of the L_1,270_ signal and the Car level and the rate of irreversible bleaching of Chl, indicating that when the normal two Cars were present the complex was less susceptible to inactivation on illumination in the presence of molecular oxygen (Telfer et al. 1994b). The fact that when two Cars were present [i.e. as seen in the native structure, Loll et al. (2005) and see Fig. 2], they could not quench all of the ^1^O_2_ formed is due to the fact that the ^1^O_2_ formed at P_680_ can diffuse in all directions within the complex and, because of the distance of the Cars from the source of the ^1^O_2_, a certain amount of damage will be done by ^1^O_2_ not scavenged by them (Telfer 2002).

## Conclusions

^3^P_680_ is inevitably formed within the PSII RC when operating in oxygenic organisms, which are also continuously evolving molecular oxygen, both at low light intensities and under high light, i.e. photoinhibitory, conditions. The P_680_ triplet thus forms ^1^O_2_ as the RC Cars are unable to quench the triplet before its reaction with ground state ^3^O_2_. ^1^O_2_ scavenging mechanisms are in place, including the two β-carotene molecules bound to the D1 and D2 RC polypeptides and, in vivo, some of the other Cars bound near the interface between the inner antenna polypeptides CP43 and CP47 close to the D1 and the D2 polypeptides, respectively (Loll et al. 2005), may well also scavenge some ^1^O_2_.

### ^1^O_2_ scavengers

^1^O_2_ that is not quenched by Car and hence escapes from the PSII core complex into the membrane will be quenched by tocopherol (Kruk et al. 2005) and plastoquinone (Kruk and Trebst 2008, Yadav et al. 2010), which is present in the thylakoid lipid membranes. Tocopherol has been implicated in protection against ^1^O_2_ damage to the membrane lipids (Kruk et al. 2005, Krieger-Liszkay and Trebst 2006). However, this is a sacrificial reaction and depends on resynthesis, using ascorbate, to restore depleted stocks of tocopherol. Inevitably some ^1^O_2_ will escape quenchers and may exit into the aqueous thylakoid lumen or stroma where it may damage proteins and nucleic acids. In the stroma, ascorbate is a good scavenger (Bisby et al. 1999) and it is usually present at high levels. It is replenished using glutathione and NADPH (Smirnoff 2000). For a discussion on scavenger effectiveness in photosynthetic systems. see Li et al. (2012).

### Relevance in vivo—photoinhibition and retrograde signaling

After the initial demonstration of formation of ^1^O_2_ by D1D2 complexes (Macpherson et al. 1993), ^1^O_2_ formation by isolated thylakoid membranes (TMs) after a high light treatment (photoinhibition) was demonstrated by Hideg et al. (1994), using a spin trapping technique, using the spin label 2,2,6,6,tetramethylpiperidine (TEMP) and EPR spectroscopy. These techniques have subsequently been used to demonstrate formation of ^1^O_2_ in PSII-enriched particles subjected to high light conditions (Krieger et al. 1998, Fufezan et al. 2002) though use of this technique in leaves is not possible (Fischer et al. 2013).

Since our initial demonstrations, during the early 1990s, of ^1^O_2_ detection in PSII RCs the techniques have been expanded greatly not only to show its involvement in photoinhibition in cells (see reviews by Krieger-Liskay et al. 2008, Fischer et al. 2013) but also it has been invoked in retrograde signaling (from the chloroplast to the nucleus) inducing cellular responses to environmental stresses including high light (op den Camp et al. 2003, Apel and colleagues; Fischer et al. 2004, Trianphylidès et al. 2009, Reinbothe et al. 2010).

Some of the new techniques employed recently, to detect ^1^O_2_, are changes in fluorescence or luminescence of ^1^O_2_-specific probes (such as DanePy and singlet oxygen sensor green, SOSG) along with imaging (for further information on the success and problems with these techniques, see Fischer et al. 2013). Fischer et al. (2007) used the DanePy technique to detect ^1^O_2_ in the cytoplasm of *Chlamydomonas reinhardtii* cells after very high intensity light stress. The significant result they found was that treatment with herbicides, which change the redox potential of Q_A_ (Rutherford and Krieger-Liszkay 2001), had the expected effect on the size of the ^1^O_2_ signal, indicating the PSII origin of the ^1^O_2_. SOSG was also used with confocal microscopy to image ^1^O_2_ formation by *Synechocystis* sp. PCC 6803 cells (Sinha et al. 2012). Additionally histidine-catalyzed molecular oxygen uptake has been demonstrated in high light-stressed *Synechocystis* sp. PCC 6803 cells (Rehman et al. 2013), and exogenously formed ^1^O_2_ (Rose Bengal sensitized) has been shown to stimulate gene expression in *Arabidopsis thaliana* (see Fischer et al. 2013).

As discussed already there are so many quenching processes going on that it was thought that it was not possible for ^1^O_2_ to get far enough to act as a signal to activate protein synthesis in the nucleus, and the debate rages as to whether ^1^O_2_ can travel that far and induce gene expression directly or whether the ^1^O_2_ detected in the cytoplasm is produced by secondary reactions. It is more likely that lipid peroxidation side products, i.e. peroxyl radicals, regenerate ^1^O_2_ by the Russell mechanism (Miyamoto et al. 2007). Recent evidence for this mechanism comes from Pospíšil and colleagues in response to both heat (Chan et al. 2012) and high light (Yadav and Pospíšil 2012).

Many calculations have been carried out to try and work out the distance ^1^O_2_ might be able to travel in the chloroplast and cell— and the projected distance is getting longer and longer. According to Moan (1990), it should only travel <70 nm before being quenched or decaying, but a more recent estimate in liposomes was >100 nm in 10 µs (Ehrenberg et al. 1998). However, it is likely that this would be much less in TMs as they contain, in addition to ascorbate, a lot of unsaturated lipids and proteins which would act as physical quenchers of ^1^O_2_, as pointed out by Ehrenberg et al. (1998). This will also be the case in the highly dense stroma and cytoplasm. More recently, Skovsen et al. (2005) showed that dye-sensitized ^1^O_2_ can travel ∼270 nm in 6 µs in the aqueous region of cells. However, this was measured in rat neurons and, as already mentioned, plant cells have high concentrations of antioxidants such as ascorbate in their cytoplasm (Bisby et al. 1999) which would reduce this distance considerably.

There is also the question of how much ^1^O_2_ is formed. The greater the amount, the more chance that a few molecules will travel some distance before meeting a quencher. Fischer et al. (2007) showed that it required very high light intensities to produce detectable levels of ^1^O_2_ in the cytoplasm of *C. reinhardtii* cells. However, it should be noted that in leaves TMs come very close to the chloroplast envelope and some chloroplasts are very close to the nucleus, although distances will still be greater than 270 nm. There is also a question of whether ^1^O_2_ originating from PSII can carry out retrograde signaling directly or whether it activates a signal transduction pathway directed to the nucleus to activate gene expression in which secondarily produced ^1^O_2_ may well play a part (Laloi et al. 2007, Baruah et al. 2009). The low light stress that also results in formation of some ^1^O_2_ in the PSII RC (Keren et al. 1997) probably only yields levels that are sufficient to be involved in photoinhibition and triggering of the turnover of the D1 protein, and not for retrograde signaling.

## Funding

This study was supported by the Agricultural and Food Research Council (AFRC) and the Biotechnology and Biological Sciences Research Council (BBSRC) [funding to Professor Jim Barber] and the Science and Engineering Research Council (SERC) and the Engineering and Physical Sciences Research Council (EPSRC) [funding to Professor David Phillips].

## Abbreviations

Car: carotenoid
EPR: electron paramagnetic resonance
L_1,270_: luminescence at 1,270 nm
LHC: light-harvesting complex
PDT: photodynamic therapy
^1^O_2_: singlet oxygen, ^3^O_2_, triplet oxygen
P_680_: primary electron donor in PSII
Pheo: primary electron acceptor in PSII
RC: reaction center
Q_A_ and Q_B_: secondary electron acceptors in PSII
RC: reaction center
RNO: *p*-nitrosodimethylaniline
ROS: reactive oxygen species
RP: radical pair
SOSG: singlet oxygen sensor green
TM: thylakoid membranes
Yz: tyrosine electron donor to P_680_^+^
